# Rationale and design of a randomized controlled trial of the effect of retinol and vitamin D supplementation on treatment in active pulmonary tuberculosis patients with diabetes

**DOI:** 10.1186/1471-2334-13-104

**Published:** 2013-02-26

**Authors:** Qiuzhen Wang, Aiguo Ma, Ib Christian Bygbjerg, Xiuxia Han, Yufeng Liu, Shanliang Zhao, Jing Cai

**Affiliations:** 1 The Institute of Human Nutrition, Medical College of Qingdao University, 38 Dengzhou Road, Qingdao 266021, China; 2 Department of International Health, University of Copenhagen, Copenhagen DK-1014, Denmark; 3 Microbiology teaching and research division, Medical College of Qingdao University, 308 Ningxia Road, Qingdao 266021, China; 4 Clinical department, Qingdao Chest Hospital, 896 Chongqing Middle Road, Qingdao 266000, China; 5 Clinical department, Lingyi Chest Hospital, East Fenghuang Avenue, Linyi 276000, China

**Keywords:** Pulmonary tuberculosis, Diabetes mellitus, Retinol, Cholecalciferol, Randomized controlled trial

## Abstract

**Background:**

The association between pulmonary tuberculosis (PTB) and diabetes mellitus (DM) has been previously attracted much attention. Diabetes alters immunity to tuberculosis, leading to more frequent treatment failure in TB patients with DM. Moreover, TB and DM often coincide with micronutrients deficiencies, such as retinol and vitamin D, which are especially important to immunity of the body and may influence pancreas β-cell function. However, the effects of retinol and vitamin D supplementation in active TB patients with diabetes on treatment outcomes, immune and nutrition state are still uncertain. We are conducting a randomized controlled trial of vitamin A and/or D in active PTB patients with DM in a network of 4 TB treatment clinics to determine whether the supplementation could improve the outcome in the patients.

**Methods/design:**

This is a 2×2 factorial trial. We plan to enroll 400 active PTB patients with DM, and randomize them to VA (2000 IU daily retinol); VD (400 IU daily cholecalciferol); VAD (2000 IU daily retinol plus 400 IU cholecalciferol) or control (placebo) group. Our primary outcome measure is the efficacy of anti-tuberculosis treatment and ameliorating of glucose metabolism, and the secondary outcome measure being immune and nutrition status of the subjects. Of the first 37 subjects enrolled: 8 have been randomized to VA, 10 to VD, 9 to VAD and 10 to control. To date, the sample is 97.3% Han Chinese and 91.9% female. The average fasting plasma glucose level is 12.19 mmol/L.

**Discussion:**

This paper describes the design and rationale of a randomized clinical trial comparing VA and/or VD supplementation to active pulmonary TB patients with DM. Our trial will allow rigorous evaluation of the efficacy of the supplementation to active TB and DM therapy for improving clinical outcomes and immunological condition. This detailed description of trial methodology can serve as a template for the development of future treatment scheme for active TB patient with DM.

**Trial registration:**

ChiCTR-TRC-12002546

## Background

The merging epidemics of pulmonary tuberculosis (PTB) and diabetes mellitus (DM) and their synergistic role in causing human disease has been attracted much attention [1]. In China, where there are experiencing incredible increase in DM prevalence, an age standardized prevalence of diabetes of 9.7% (10.6% in men and 8.8% in women) and prevalence of pre-diabetes as 15.5% (16.1% in men and 14.9% in women) [2], also a high burden of PTB exists. The combination of tuberculosis and diabetes mellitus and double burden of TB and DM represent a big health threat, which make the treatment failure more frequent and result in more community acquired TB infection. Diabetes alters immunity to tuberculosis, which will lead to higher mycobacterial burden and longer time to culture conversion with treatment, and a higher rate of relapse might result. In studies assessing time to sputum-culture conversion, diabetic patients seem to take longer time to achieve culture negativity. Guler et al. reported that patients with diabetes who received tuberculosis treatment had longer sputum-culture conversion times than non-diabetic patients (67 *vs* 55 days; p=0.02) [3]. Similarly, Restrepo et al. [4] used survival analysis to measure time to culture conversion, and found median time to culture negativity was significantly longer in diabetic patients than that in controls. Radiographic findings in tuberculous patients with diabetes differ from pure TB patients. It was widely believed that pulmonary tuberculosis patients with diabetes presented with an atypical radiographic pattern and distribution and lower lung field tuberculosis was seen more frequently in TB patient with DM. Overlapping toxicities and various drugs used may worse diabetic control and alter the pharmacokinetics of antituberculosis drugs, which makes the clinical treatment of TB patients with DM more difficult.

TB and diabetes often coincide with malnutrition. TB patients frequently suffer from deficiencies of nutrients, such as vitamins A and D, which are fundamental to the integrity of the immune response, especially the host immune response toward Mycobacterium. Studies have shown that vitamin A has an immune protection role against human tuberculosis. In addition, vitamin A supplementation results in a modulation of the immune response in patients with tuberculosis [5,6]. The classic role of vitamin D in the regulation of calcium and phosphate metabolism, and its effects on bone health, have been well established. More recently, a critical role of vitamin D in immunity, respiratory health and insulin resistance has been proposed [7,8]. Low serum vitamin D has been discovered to be associated with higher risk of active tuberculosis. Researches revealed that vitamin D plays a key role in the production of a molecule called cathelicidin, which kills the mycobacterium tuberculosis organism [9,10]. Furthermore, a double-blind randomized controlled trial conducted in 192 healthy adult TB contacts in London discovered that a single oral dose of 2.5 mg vitamin D significantly enhanced the ability of participants' immunity to mycobacteria [11]. However, a trial in Guinea-Bissau found no effect of vitamin D (100,000 IU of cholecalciferol at inclusion and again 5 and 8 months after the start of treatment) on clinical outcome and mortality in patients with TB [12].

Furthermore, vitamins play an important role in glucose metabolism. It has been reported that vitamin D can regulate the β cell function in pancreatic islet, the insulin activity and the systematic inflammation level. Plasma 25(OH) D levels are positively association with both β-cell function and insulin sensitivity [13]. A trial carried out in USA found that in adults at risk of type 2 diabetes, short-term supplementation with cholecalciferol improved β cell function and had a marginal effect on attenuating the rise in Hb A(1c) [14]. Also, Vitamin A and its metabolites, retinoids, have emerged as key participants in the mechanisms of regulation of cellular metabolisms and energetics, which may also play a key role in the process of glucose metabolism.

We designed this VA/D supplementation trial to test the efficacy of micronutrient intervention on active TB patients with DM. We are implementing this randomized controlled trial in a network of TB therapy clinics that offer TB treatment by primary care providers, including standard DM treatment. The primary objective of our trial is to determine whether VA, VD or VAD supplementation is efficacious on anti-tuberculosis treatment and insulin resistance. The secondary objective is to investigate the underlying mechanism including immunological function, nutritional status, etc. We here provide a detailed methodological description of this trial, which can serve as a template for other investigators designing the comprehensive treatment of TB and DM. In addition, we discuss the efficacy of VA and/or VD supplementation on TB and DM to guide future clinical treatment. Finally, we report baseline characteristics of our recruited study population.

## Methods/design

### Study setting

The VA/D supplementation trial is being conducted in a network of 4 TB clinics in Qingdao and Linyi city in Shandong province in China. Together, these clinics provide care for approximately 5,000 active pulmonary tuberculosis patients per year and nearly 10% have DM. These four clinics are the sites for study recruitment, delivery of the VA and/or VD intervention, and all research visits.

VA and/or VD supplementation is delivered by the physicians, together with the nurses, social workers to take care of the patients. The medical team provides comprehensive medical care, including TB and DM pharmacotherapy.

### VA/D supplementation scheme

This is a 2-by-2 factorial-design intervention trial. Active pulmonary tuberculosis patients with diabetes mellitus are offered onsite TB and DM evaluation and treatment by primary care providers, and are free to choose whether to receive VA/D supplementation. Primary care providers receive special training by the project team, including the related knowledge of TB and DM, especially the association between TB and DM; the related nutritional deficiencies and the role of micronutrients in cell-mediated immunity.

Once sign the informed consents, the patients will be randomly assigned to VA (2000 IU daily retinol on top of normal pharmacotherapy) or VD (400 IU daily cholecalciferol on top of normal pharmacotherapy) or VAD (2000 IU daily retinol plus 400 IU daily cholecalciferol on top of normal pharmacotherapy) or control (placebo on top of normal pharmacotherapy) group.

#### Trial inclusion and exclusion criteria

Potential trial subjects are eligible for inclusion if they are affected with active pulmonary tuberculosis and have fasting plasma glucose (FPG) of ≥126 mg/dL or 2-hour glucose level of 200 mg/dL or above by OGTT; have no history of previous anti-tuberculosis treatment and oral hypoglycemic drugs or insulin used; plan to initiate onsite anti-tuberculosis and DM treatment at the TB clinic within the next six months; attend their TB clinic after hospitalization once or twice per month to receive supplementation pill and physical examination; plan to stay in local place for 2 years. Participants are excluded if they are unable or unwilling to provide informed consent; are drug resistant at baseline or during the follow up; pregnancy; lactation; use of corticosteroids or supplements containing vitamin A or vitamin D during the previous month; chronic renal failure as indicated by elevated serum uric acid or creatinine concentrations; and clinical signs of neoplasm and congestive heart failure.

### Approvals and data safety and monitoring

The trial was approved by Ethics Committee of the affiliated hospital of Medical College of Qingdao University. All participants provide written informed consent. Besides of VA and/or VD supplementation, we are giving nutritional and behavioral suggestion and instruction that is highly integrated with normal clinical care to all the subjects. Also, we established a data safety and monitoring plan, which requires interim analyses after every twenty patients are enrolled.

### Recruitment

Medical providers in the four TB clinics are asked to invite all patients initiating on-site anti-TB and DM treatment to consider enrolling in the trial. Subjects also are referred by other subjects or through the project group. The initial steps in subject recruitment include a brief screening survey, informed consent, and in depth verification of eligibility using medical records and discussion with providers.

### Randomization

Random-number tables are used to allocate subjects to the VA intervention arm, VD arm, VAD arm or the placebo control arm.

### Baseline assessment

The baseline assessment consists of an interview and physical measurement. Baseline fast blood glucose, results of urine toxicology tests, liver function test and other related clinical indexes are obtained by chart review. The baseline interview is conducted prior to vitamin supplementation initiation, and at this visit all subjects are invited to attend VA/D supplementation trial. Following completion of the baseline assessment, participants receive their assignment to one of the four arms.

### Visit schedule and measures

Frequency and length of research visits during the 24 to 32 weeks (according to the anti-tuberculosis drug treatment period) intervention period are the same for subjects in each arm. We standardized the visit schedule to control for any improvement in adherence, possible side effects which might result from VA/VD supplementation alone. We use several measures to assess VA/VD adherence: self-report, pill count, and nursing records of directly observed doses and returned pill boxes.

### Treatment-related side effects

A treatment side effect scale (which measures the degree of VA and/or VD treatment-related side effects) is administered at each monthly visit, and the results are given to medical providers. Side effects include fatigue, irritability, anorexia, headache, fever, insomnia, nausea, vomiting, and other common side effects.

### Sample size and power calculations

We calculated the sample size on the basis of literature research in the related domain and analyzing the objective and indexes in this trial by using formula:

$$N={\left[\left(\left(Z_{\alpha /2}+Z_{\beta }\right)\sigma \right)/\delta \right]}^{2}$$

$$\alpha =0.05\;{\left(Z_{\alpha /2}=1.645\right)}_{,}1-\beta =0.90\;\left(Z_{\beta }=1.282\right)$$

We calculated the needed sample size for the main indexes and selected the largest one. We will need 84 subjects in each arm to have 90% power to show significant difference among trial arms. Considering participants’ compliance, none response and withdraw, a sample size of 100 per arm is needed.

### Planned statistical analyses

We hypothesize that effect of anti-TB and DM treatment in VA and/or VD arms will be better than the effect in the control arm. Descriptive statistics will be calculated and checked for balance in each arm in demographic, physical and other related measurements at baseline. Comparisons among the groups of pulmonary pathological damage, CD_4_^+^ T cell, HOMA-IR, etc., will be performed by Chi-square (categorical variables), F test (continuous variables, normal distribution) or Kruskal–Wallis H test (ranked data). Linear mixed-effects models will be used to analyze the main influencing factors.

We will also calculate anti-tuberculosis and diabetes treatment results, nutritional and immunologic indexes at three time points: before the supplementation (at the inclusion), 2 months after the supplementation, and at the end of the trial (6 to 8 months after the supplementation).

### Study sample characteristics

**Figure 1:**
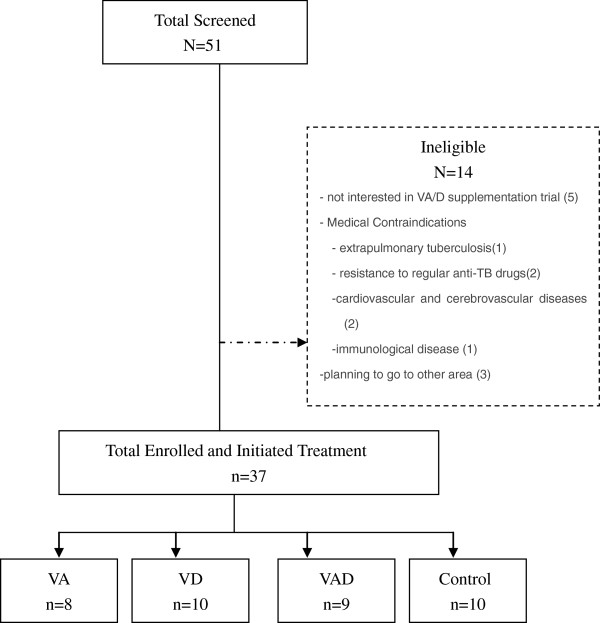
Flow chart of study recruitment and enrollment.

Screening and baseline interviews began in Oct, 2012, and are ongoing. We here present the baseline characteristics of the first 37 subjects who have been enrolled and initiated on-site anti-TB and DM treatment: Of these, 8 were randomized to the VA arm, 10 to VD arm, 9 to VAD arm and 10 to the control arm (Figure 1).

The sample is 91.9% male, 97.3% ethnic Han, with a mean age of 52 (Table 1). 83.8% subjects have education level of senior high school or lower, 75.7% married, 48.6% employed. 75.7% subjects’ BMI were normal(18.5~24.99)with one patient being severe wasting, 5 patients being mild wasting and 3 patients being overweight.

**Table 1:** Baseline characteristics of study sample (n =37)

**Sociodemograpic**	
Age, mean (sd)	52 (12.0)
Gender, n (%)	
Male	34 (91.9)
Female	3 (8.1)
Race, n (%)	
Han	36 (97.3)
Other	1(2.7)
Education, n (%)	
Senior high school or lower	31 (83.8)
Junior College and college	6 (16.2)
Marriage Status, n (%)	
Married	28 (75.7)
Widowed/divorced/single	9 (24.3)
Employed, n (%)^a^	
Employed	18 (48.6)
Retired/Unable to work/unemployed/other	16 (43.2)
**PTB-related Admission Symptoms**, n (%)	
Cough+	35 (94.6)
Cough-	2 (5.4)
Expectoration+	33 (89.2)
Expectoration-	4 (10.8)
Hemoptysis+	11 (29.7)
Hemoptysis-	26 (70.3)
Fever+	18 (48.6)
Fever-	19(51.4)
Weak+	28(75.7)
Weak-	9(24.3)
Night sweat+	19(51.4)
Night sweat-	18(48.6)
Thoracalgia+	8(21.6)
Thoracalgia-	29(78.4)
Chest stufly+	20(54.1)
Chest stufly-	17(45.9)
**PTB-related CT pathology**, n (%)	
Pulmonary cavity +	27(73)
Pulmonary cavity –	10(27)
Pulmonary infiltration+	1(2.7)
Pulmonary infiltration-	36(97.3)
**Admission Biochemical index**	
FPG, mmol/L(mean,sd)	12.19, 3.30
LDL-C, mmol/L (mean,sd)	2.42, 0.63
TC, mmol/L (M, interquartile range)	4.29, 0.85
TG, mmol/L (M, interquartile range)	1.66, 0.79
HDL-C, mmol/L (mean,sd)	1.21, 0.29
**BMI**^**b**^, n (%)	
<16	1(2.7)
16~16.9	0 (0)
17~18.4	5(13.5)
18.5~24.99	28(75.5)
25~29.99	3(8.1)
>30	0(0)

^a^ missing data.

^b^<16: severe wasting; 16~16.9: moderate wasting; 17~18.4: mild wasting; 18.5~24.99: normal; 25~29.99: overweight; ≥ 30: obesity.

At baseline, 94.6% of the sample had cough, 89.2% had expectoration, 29.7% had hemoptysis, 48.6% had fever, 75.7% had weak, 51.4% had night sweat, 21.6% had thoracalgia, 54.1% had chest stufly. As to pulmonary pathology inspected by CT, 73% of the sample had pulmonary cavity, 2.7% had infiltration. Admission biochemical examine showed that the mean fast plasma glucose concentration was 12.19 mmol/L, mean LDL-C level was 2.42 mmol/L, median TC concentration was 4.29 mmol/L, median TG concentration was 1.66 mmol/L, mean HDL-C level was 1.21 mmol/L.

Our intervention is feasible and can be implemented in a busy clinical setting. Staff at the four clinics worked collaboratively with the study team, and to date 37 participants have been enrolled from the four clinics. Health care providers communicate well with the medical director and research assistants, and nurses at each clinic record daily events on study calendars. To date, a total of 51 subjects have been screened (Figure 1).

The VA/D program is acceptable to our patients. To date, 88% of all patients who were eligible for receiving VA/D supplementation and met study inclusion and exclusion criteria have chosen to participate. No participants have refused their randomization.

The core components of our VA/D program do not require additional staff over existing clinic operating models. We will provide subsidies for medical providers, nurses, or support group facilitators on support of the Natural Science Foundation of China (NSFC) and Danone Nutrition and Education fund. We pay the VA and VD supplements used for the study.

## Discussion

To our knowledge, this study represents the first randomized controlled trial of a directly observed VA/D supplementation program in active pulmonary tuberculosis patients with diabetes that focused on the contribution of anti-TB and DM treatment effect and the possible mechanisms.

Tuberculosis and diabetes interact with each other, which might make clinical treatment become more difficult than the single disease of pulmonary tuberculosis or diabetes. As diabetes alters immunity to tuberculosis, longer times to culture conversion with treatment, and a higher rate of relapse might result. Treatment failure and death are more frequent in diabetic patients than patients with PTB alone. Alisjahbana reported that in patients with high adherence to treatment, 6-month sputum cultures were positive in 22.2% of patients with diabetes mellitus and in 6.9% of controls and these differences remained after adjustment for age, sex, body mass index, and other factors [15]. In a case–control study [16] treatment failure or death was seen in 41% of the patients with tuberculosis and diabetes mellitus, but in only 13% of those with tuberculosis alone. A recent study by Wang and colleagues [17] discovered that 1-year all-cause mortality was 17.6% in diabetic PTB patients versus 7.7% in non-diabetic controls, and death specifically attributable to pulmonary tuberculosis was significantly more common in diabetic patients (12.2% vs 4.2%).

The most important effector cells for containment of tuberculosis are phagocytes, monocytes and lymphocytes. Diabetes is known to affect chemotaxis, phagocytosis, activation, and antigen presentation by phagocytes in response to M tuberculosis. Vitamin A is an essential micronutrient for a variety of physiological processes, such as tissue differentiation, immunity, and vision. Also, the impacts of VA and retinoids on energy metabolism in animals and humans have been demonstrated in some basic and clinical investigations [18]. Also, it has been proposed recently that vitamin D play a critical role in immunity, respiratory health and insulin resistance. Vitamin A, its metabolites and vitamin D directly inhibit mycobacterial growth in culture [19].

A recent randomized trial of vitamin A and Zinc supplementation focused on the effect on treatment outcomes in TB patients and found that supplementation with vitamin A and zinc did not affect treatment outcomes in participants with pulmonary tuberculosis at 8 wk [20]. An important difference between this trial and our study is that single dose of 200,000 IU retinyl palmitate was used in the reported trial, while daily supplementation of 2000 IU retinol is being supplemented in our study. Also, vitamin A and Zinc supplementation on serum fasting blood sugar, insulin, apoprotein B and A-1 in patients with type I diabetes has been carried out [21], and found that combined zinc and vitamin A supplementation can improve serum apoprotein A-I, apoprotein B and the apoprotein B/apoprotein A-I ratio in patients with type I diabetes.

Some randomized controlled studies have focused on effect of vitamin D on glucose indices and vascular markers in type 2 diabetes[22,23], which found that Vitamin D supplementation attenuated the increase in glycemia, and increased insulin secretion, improved systolic blood pressure and B-type natriuretic peptide levels. Mitri et al. [14] implemented a 2-by-2 factorial-designed, double-masked, placebo-controlled trial and discovered that cholecalciferol (2000 IU once daily) supplementation for 16 wk can improved β cell function and had a marginal effect on attenuating the rise in Hb A(1c) in adults at risk of type 2 diabetes.

Vitamin D has been shown to be involved in the host immune response toward Mycobacterium tuberculosis. Recently several VD supplementation trials to active TB patients are available. Administration of four doses of 2.5 mg vitamin D3 significantly hasten sputum culture conversion in patients with the *tt* genotype of the *Taq*I vitamin D receptor polymorphism [24]. However, C Wejse et al. [12] reported that supplementation of three doses of 2.5 mg vitamin D3 at baseline, 5 months, and 8 months did not influence clinical severity score or time to sputum smear conversion in 365 patients in Guinea Bissau. It is possible that the dose used was insufficient. In our trial, we are supplementing VD by daily dose of 2000 IU cholecalciferol, and we are measuring the effect of the supplementation on clinical treatment in active TB patients with diabetes. In addition, prior studies have found that supplementation of VA or VD is associated with improved β cell function or hasten sputum culture conversion, our trial will allow us to determine whether this association will remain true for VA and/or VD use in patients with both TB and DM who have more severe clinical symptom and poorer prognosis, and the underlying mechanism is being investigated.

Despite its strengths, trial limitations should be noted. In our trial, only some doses of VA and VD are observed. However, we designed these doses on the basis of acquiring important information including the VA nutrition status in China by national survey [25], the recommended VA and VD intake formulated by the Chinese nutrition society. Also, abundant literature researches concerning the VA, VD level in the supplementation trials have been implemented. In addition, different distribution of some aspects of the subject in the four arms such as severity of the disease, fundamental nutrition status, socioeconomics status etc. may interfere or reduce our measured effect size. To minimize the differential effect between the four arms, we balanced the four arms regarding age, sex and randomized the subjects to different groups. Furthermore, multivariate logistic regression will be used to identify the measured size to adjust the possible impact factors.

We expect that results of our VA and/or VD supplementation trial will advance our knowledge of the efficacy of directly observed therapy for active PTB and DM. If our results provide evidence that VA and/or VD supplementation is effective in PTB and DM, further research protocols can be developed to observe the efficacy of other kind of micronutrients related to glucose metabolism and/or anti-mycobacterium on the patient with both PTB and DM. Data from this study will also allow us to examine the optimal biomarkers associated with PTB and DM treatment influenced by micronutrients. Newer regimens for PTB and DM treatment will emerge which may improve outcomes of clinical therapy and/or shorten the treatment period.

## Abbreviations

PTB: Pulmonary tuberculosis;DM: Diabetes mellitus;VA: Vitamin A;VD: Vitamin D

## Competing interests

The authors declare that they have no competing interests.

## Authors’ contributions

QW developed and implemented the study protocol, oversaw research assistants, wrote the initial and final drafts of the manuscript, and obtained a smaller portion of the funding for the study. AM obtained most funding for the study and oversaw the study’s conduct, developed the study protocols and provided guidance on study design and methods. ICB directed the study protocols and provided guidance on study design and methods. XH provided guidance on study design especially about the laboratory test of pulmonary tuberculosis. YL and SZ assisted in the development of study protocols. JC managed study data. All authors have read, contributed to, and approved the manuscript.

## Authors’ information

QW is an associate professor in Nutrition Institute in Qingdao University. She has specialized in epidemiology and nutrition. AM, a senior dietitian, is the head of the Institute of Human Nutrition, Medical College of Qingdao University and he is also a routine director of the Chinese Nutrition Society. ICB is a senior professor of Institute of International Health, University of Copenhagen, Copenhagen, Denmark. He is a specialist in tropical medicine and in infectious diseases. His research interests are double burden of communicable & non-communicable diseases, malaria, TB, HIV, diabetes, and their interactions. XH is a senior expert in epidemiology. YL and SZ are clinical doctors specified in TB who have abundant clinical experience. JC is a PhD candidate in the Institute of Human Nutrition, Medical College of Qingdao University, Qingdao, China.

## Pre-publication history

The pre-publication history for this paper can be accessed here:

http://www.biomedcentral.com/1471-2334/13/104/prepub
